# Survival outcomes of unilateral retinoblastoma based on pathological risk stratification-experience at a tertiary care centre in Pakistan

**DOI:** 10.3332/ecancer.2022.1360

**Published:** 2022-03-01

**Authors:** Najma Shaheen, Naila Inayat, Sehar Bashir, Umer Nisar Sheikh, Muhammad Abu Bakar, Palwasha Rehman

**Affiliations:** 1 Department of Pediatric Oncology, Shaukat Khanum Memorial Cancer Hospital and Research Centre, 7A, Johar Town, Lahore 54782, Pakistan; 2 Department of Pathology, Shaukat Khanum Memorial Cancer Hospital and Research Centre, 7A, Johar Town, Lahore 54782, Pakistan; 3 Epidemiologist and Bio-statistician-Cancer Registry, Shaukat Khanum Memorial Cancer Hospital and Research Centre, 7A, Johar Town, Lahore 54782, Pakistan; a https://orcid.org/0000-0002-3745-380X

**Keywords:** children, enucleation, retinoblastoma, pathology

## Abstract

Retinoblastoma (RB) is the most common childhood intraocular malignancy. In high-income countries over the past decade, upfront enucleation for unilateral RB is least favoured due to other alternatives that can help in globe preservation, but in low-middle income countries it is still the preferred option due to lack of resources and expertise. The treatment of RB after enucleation is tailored based on the histopathological risk features, as adjuvant chemotherapy with high-risk features reduces the risk of metastasis. The aim of our study was to analyse the survival outcomes of adjuvant therapy based on histopathological risk stratification in patients who underwent upfront enucleation for unilateral RB with advanced disease. A retrospective study was carried out at Shaukat Khanum Memorial Cancer Hospital and Research Centre, Pakistan. A total 113 patients (aged 3 months till 16 years) diagnosed with unilateral RB who had upfront enucleation from July 2009 till January 2019 were included in this study. The mean age of diagnosis was 37.4 months (±24.5) and male-to-female ratio of 1.3:1. The most common clinical presentation was leukocoria (74.3%). Patients who underwent enucleation had advanced disease; group D present in 62.8% followed by group E (32.7%). Histopathology revealed high-risk features in 29 patients (25.7%) and intermediate risk in 54 patients (47.8%). Disease progression and relapse was seen in patients with high-risk histopathological features. The 4-year over-all survival and EFS observed for this cohort was 74% and 71%. Awareness about the early symptoms among the general population and health care personnel at a nationwide level is needed to facilitate early detection and lessen disease related morbidity and mortality.

## Introduction

Retinoblastoma (RB) is the most common intraocular malignancy in childhood and accounts for about 2% of all childhood cancers [1]. The incidence of RB ranges from 1 case per 15,000–20,000 live births worldwide [2]. RB occurs due to germline or somatic mutations. Loss of function of RB 1 gene on chromosome 13q results in the germline (hereditary) RB, which commonly presents as bilateral eye involvement in early age group. Somatic RB (non-heritable RB) results from loss of function of RB1 gene in the retina; patients will usually have unilateral, unifocal disease and present at a later age compared to the heritable type [3].

In high-income countries, due to early detection and optimal health facilities, the overall survival (OS) of RB has increased to more than 95%; hence, the aim of treatment has been shifted to globe salvage [4]. In low-middle income countries (LMICs), low-socioeconomic status, advance disease and abandonment are the main risk factors leading to poor outcomes among RB patients [5, 6]. Asia-pacific region contributes significantly to RB; almost 43% of the children diagnosed globally belong to this region. India ranking in the first place where 1,500 cases are detected annually, whereas Pakistan stands at the fourth place [7]. The annual crude incidence of RB in children under the age of 5 and 10 years in Karachi (Pakistan) was reported to be 4.0/100,000 and 2.4/100,000 [8].

Majority of the intraocular disease (small or medium sized) can be treated with globe preserving strategy, however primary enucleation remains the treatment of choice for advanced disease [9]. RB can be stratified based on clinical presentation and histopathological features. Clinical features such as irreversible neovascular glaucoma, aseptic orbital cellulitis (group E disease) is associated with high-risk histopathological features and require adjuvant chemotherapy after enucleation as there is always an increased risk of metastasis [10, 11].

In high-income countries, the incidence of histopathological high-risk features after enucleation had declined over the years due to early diagnosis and referral; however, cases are still reported from LMICs like Pakistan [12, 13]. Post enucleation histopathological features play an important role in assessing the disease severity, results from high-income countries cannot be generalized as presentation of disease differs in LMICs [14]. The purpose of our study was to analyse the survival outcomes of adjuvant therapy based on histopathological risk stratification in patients who underwent upfront enucleation for unilateral RB with advanced disease.

## Materials and methods

### Patients and diagnostic evaluation

Shaukat Khanum Memorial Cancer Hospital and Research Centre is among one of the leading cancer care charity hospitals of the country, where oncology patients (adults/children) receive treatment irrespective of their ability to pay. It is 195 beds (in-patient) facility and approximately 500–550 paediatric oncology patients are registered annually.

Hospital-based electronic data of children with RB from July 2009 to January 2019 was reviewed after approval from the institutional review board. A total of 113 children with unilateral RB were identified who had upfront enucleation and were naive for chemotherapy.

RB diagnosis was made either by indirect ophthalmoscopic examination under anaesthesia (EUA) or on histopathology confirmation after enucleation [15]. Extent of disease evaluation included ocular EUA, B scan (where applicable), magnetic resonance imaging scan or contrast enhanced computerised tomography scan of brain and orbit in all patients. Cerebrospinal fluid examination with cytospin, bone marrow biopsy and aspiration with immunocytology were done for disease staging [16]. According to International Retinoblastoma Classification (IIRC), the intraocular disease was grouped [17]. All cases were subjected to histological examination for tumour grade/differentiation and evaluation of high-risk features, which included the presence and level of optic nerve involvement, post-laminar and extension to the optic nerve resection margin, location and extent of invasion of uveal tract. Invasion was measured in millimetres and labelled as massive if more than 3 mm in diameter. Other histological features such as necrosis, calcification, involvement of other ocular structures; anterior chamber, ciliary body sclera was also noted [18].

### Treatment plan and follow-up

Adjuvant chemotherapy was offered based on histological features, patients with low-risk features (prelaminar invasion, focal choroidal invasion (<3 mm) and ciliary body involvement) were offered no chemotherapy. Those with intermediate-risk features (massive choroidal invasion with >3 mm, and anterior chamber invasion) were offered four courses of chemotherapy and patients with high-risk features (optic nerve cut end and scleral invasion) were given six courses of chemotherapy [19]. The chemotherapy protocol (vincristine 1.5 mg/m^2^, carboplatin 600 mg/m^2^ and etoposide 300 mg/m^2^) was given 21 days apart with the neutrophil count ≥1× 10^9^/L and platelets ≥100 × 10^9^/L. External Beam Radiotherapy at a dose of 40 Gy in 20 fractions was offered to patients with optic nerve cut end invasion [20]. Post treatment patients had 3 monthly follow-up for 2 years and then after every 6 months till the age of 10 years.

### Statistical analysis

Statistical analysis was performed using the Statistical Package for the Social Sciences version 20. Mean and standard deviation were reported for quantitative data like age and frequency and percentages were reported for qualitative data like; sex, presenting signs and IIRC Group. OS and event free survival (EFS) was calculated using Kaplan–Meir curve method. The EFS was calculated from the date of diagnosis until date of death or date of progression of disease or date of relapse. OS was calculated from date of diagnosis till date of death. Log rank was used to calculate the survival difference and *p*-value of ≤0.05 was considered to be statistically significant.

## Result

### Patient characteristics

A total 113 patients were included in this report. The mean age of diagnosis was 37.4 ± 24.5 months, with male-to-female ratio of 1.3:1. The median follow up time was 34.9 months (SD ± 27.8) (Interquartile range (IQR)- 10–62). The eldest patient in our group was 15 years old. Eighty-two patients (72.6%) belonged to urban areas, whereas 6.2% patients were referred from neighbouring country (Afghanistan).

The most common clinical presentation of disease was leukocoria (74.3%), and the most common clinical group seen was group D (60.2%) (Table 1) in this report. Seventy-two patients (63.7%) had upfront enucleation and one child had exenteration from outside facility. Forty patients (35.4%) underwent enucleation at our institute. The mean number of days from the enucleation to start of chemotherapy was 25.3 ± 26.4 days (range 0–130 days).

### Outcomes

In our cohort, advance disease on presentation (group D and E) had intermediate and high-risk features on histopathology requiring adjuvant chemotherapy with *p* value of 0.02.

Twenty-six enucleated (23%) eyes had well differentiated tumour histology, 31 eyes (27.4%) had moderately differentiated histology, whereas 53 eyes (46.9%) had poorly differentiated histology. Thirty patients (26.5%) were stratified as low risk based on histopathological features of enucleated eye, 54 patients (47.8%) as intermediate risk, whereas 29 patients (25.7%) had high-risk features. Intermediate and high-risk patients were offered chemotherapy, whereas radiation therapy was only offered to patients stratified as high risk.

The low-risk group (*n* = 30) had good outcomes; relapse disease was seen in one patient only. Disease progression and relapse were seen in the groups having intermediate and high-risk features on histopathology. Among the intermediate risk group, one patient and three patients with high-risk histopathological features died during treatment due to sepsis (Table 2).

At the end of treatment, complete remission was achieved in 83 patients (73.5%). Death as first event was observed in four patients (3.5%). Disease progression was documented in seven patients (6.2%), among them three patients refused further treatment, whereas four patients died due to disease burden. Disease relapse was reported in 12 patients (10.6%). The average time of relapse noted after the end of treatment was 10 months (range 2–36 months). Seven patients had intracranial relapse; palliative radiation therapy was given to them. Leptomeningeal metastasis was seen in three patients. One patient had pineal blastoma along with spinal metastasis 18 months after end of therapy, whereas one patient had extraocular relapse. Unfortunately, seven patients (5.3%) abandoned therapy during their course of treatment.

**Figure 1.**
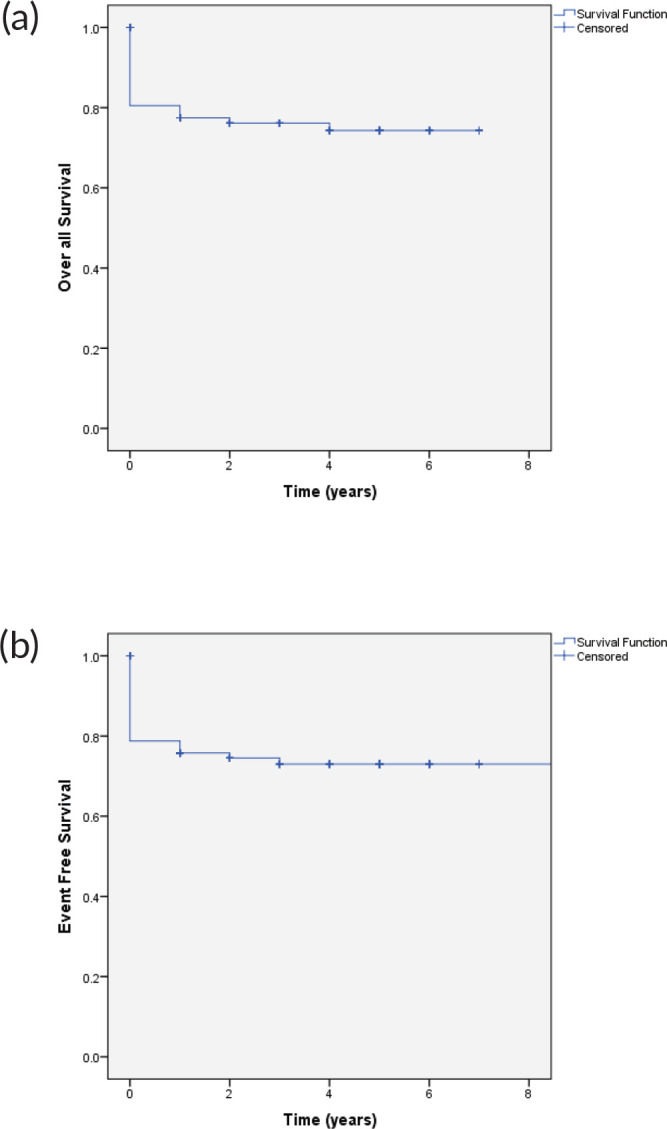
(a): OS and (b): EFS.

**Figure 2.**
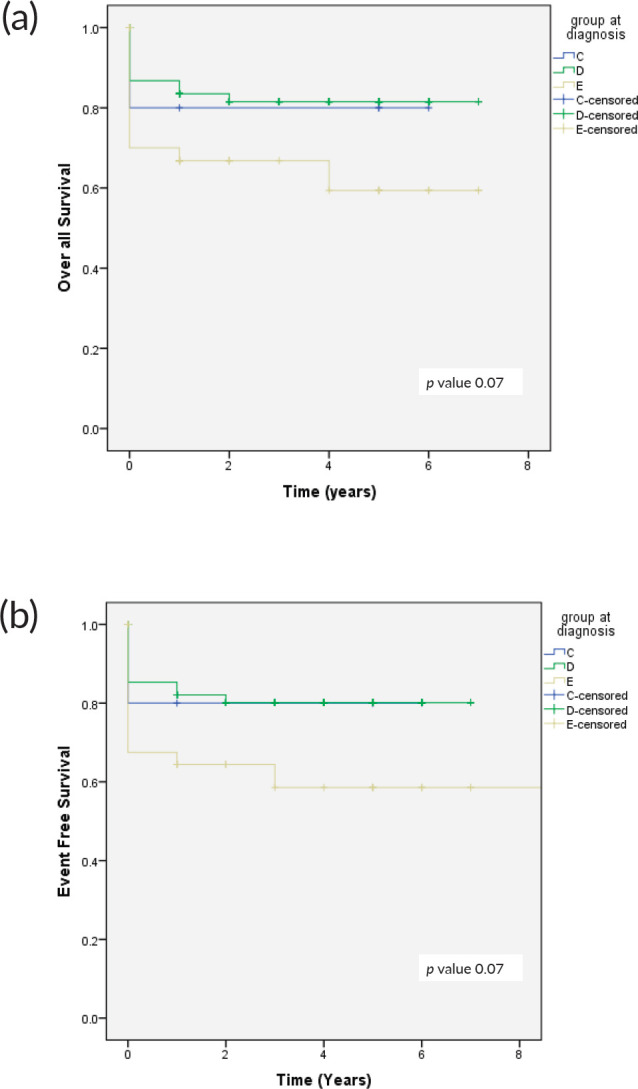
(a): OS in relation to clinical group and (b): EFS in relation to clinical group.

The 4-year OS and EFS observed for this cohort was 74% and 71%, respectively (Figure 1). OS of patients with group C at 4 years was 80%, group D 82.4%, and group E was 65%, respectively. The event-free survival in group C, D and E was 80%, 79%, and 59%, respectively (Figure 2).

**Figure 3.**
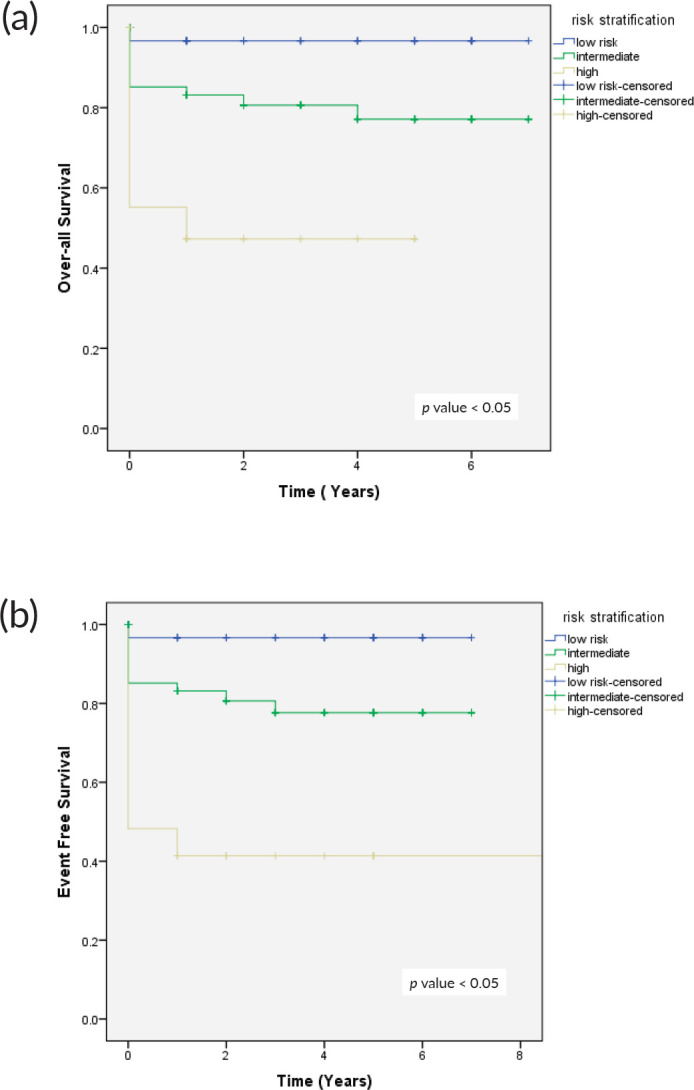
(a): OS as per risk stratification and (b): EFS as per risk stratification.

The 4-year OS survival based on histopathological risk stratification in low-risk group was 96.7%, in intermediate risk group 79.6% and 43% in high-risk group, whereas 4-year EFS in low-risk group was 96%, 76% in intermediate risk and 41% in high-risk group respectively (Figure 3).

## Discussion

Asia-pacific region of the world contributes significantly to the diagnosis of RB, Pakistan is among the top ten countries of the regions that shares significant burden of this disease [21]. The clinical presentation of the RB is variable including leukocoria, strabismus, vision loss or painful eye [22]. Leukocoria was the most common clinical presentation (74.3%) seen in our patients followed by proptosis, comparable to other LMICs [23]. Along with leukocoria, proptosis and vision loss were the usual presenting clinical signs associated with advanced group disease D and E (*p* value 0.002). The mean age of presentation among our cohort was 37.4 months, which is higher than our neighbouring countries where it was reported to be 20 months [24]. This may reflect delay in diagnosis and referral of our study population to the cancer treatment centre. A male predominance was seen in our patients, other studies from Asia have reported similar results, although no sex predilection has been seen in RB and the differences mainly can be due to gender discrimination [25, 26].

The advanced group D and E disease among our patients had high-risk histopathological features and required adjuvant chemotherapy. Optic nerve cut end invasion termed as high-risk feature was present in 25.7% of patients and they received radiation therapy along with adjuvant chemotherapy. Globe salvage is being promoted for group D eyes with the introduction of the new treatment modalities like intra-arterial chemotherapy (IAC), but disease relapse can still not be ignored. However, for group E disease primary enucleation is still the treatment of choice [27]. Unfortunately, in our setting we lack the expertise of administration of IAC.

**Figure 4.**
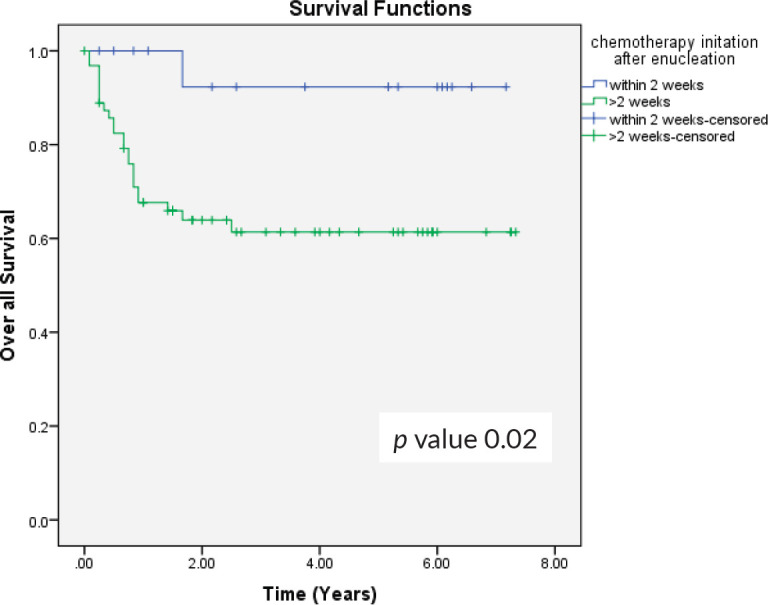
OS in relation to the initiation of chemotherapy after enucleation.

Previous study has shown that timely enucleation followed by adjuvant chemotherapy in high-risk histopathology reduces the risk of metastatic disease [28]. In our data, 15 patients had disease progression and metastasis in intermediate and high-risk group who received adjuvant chemotherapy after 2 weeks of enucleation. When we analysed the outcomes based on initiation of adjuvant chemotherapy, the patients who received chemotherapy within 2 weeks of enucleation had better survival compared to those who received after 2 weeks (94.1% versus 64.1%, *p*-value 0.02) (Figure 4).

The histopathological feature of RB plays major role in predicting outcome of the disease [29]. On microscopic level, RB reveals small hyperchromatic cells with high nuclear to cytoplasmic ratio, the tumour is differentiated into well differentiated to poorly differentiated (based on the Homer–Wright rosettes) [30]. In our data, 62 patients (54.8%) with poorly and moderately differentiated histology had high risk features and were given adjuvant chemotherapy. Disease relapse and progression was also seen among these patients, although statistically it was not significant (*p* value- 0.26). Kashyap *et al* [31] reported that poorly differentiated tumours are associated with multiple high-risk features, as compared to well differentiated tumours (*p* value of <0.001).

Early diagnosis and treatment can improve outcomes in RB. In our cohort, disease progression was seen in 6.2% patients while on treatment, which is within the estimated range for LMICs [32]. Slightly better outcomes were seen in patients who had enucleation at our facility as compared to those who had at outside facility (EFS 75.3% versus 70%).

Five patients (4.4%) in our study had group C disease on EUA, but they underwent upfront enucleation (at an outside facility). Majority of our patients (72.6%) that presented with advanced disease belonged to urban areas. Due to lack of expertise and limited resources in our country, novel therapies for globe salvage could not be offered, it can be speculated that the treatment option of upfront enucleation can be one of the risk factors that lead to advanced disease presentation. Since it is retrospective study exact risk factors that lead to advance disease could not be assessed.

In our report, seven patients (5.3%) abandoned treatment, even though free of cost therapy was offered at our centre. Abandonment had always been a challenge when treating oncology patients, high rate of abandonment has been seen in RB patients in LMICs, intensive chemotherapy regimens and mutilating surgical procedures such as enucleations and prolong follow up can be considered as a risk factor [33]. In the central American Association of Pediatric Hematology Oncology II trial, 102 patients (*n* = 161) received upfront enucleation and 59 patients underwent delayed enucleation. Although they were successful in reducing the abandonment in patients with advanced disease, the children with upfront enucleation had better outcomes as compared to those with delayed enucleation (5-year OS 94% versus 74%: *p* value < 0.001) [34].

This study is retrospective with some inherited limitations, as data was collected through online electronic media and majority of our patients were referred after enucleation, the exact onset of symptoms could not be determined. Secondly, to determine the intraocular disease group in children, we relied on the examination done by referring ophthalmologist. In resource limited setting, patients with advanced unilateral disease (group D or E) without treatment modalities for globe salvage upfront enucleation can be considered. RB awareness programs for early recognition and referrals, multiple disciplinary team (MDT) meetings with experts or telemedicine clinics are the ways through which management of RB could be implemented in LMICs, resulting in early recognition and reducing mortality rates [35]. We have started similar program as National RB MDT under guidance of Pakistan Society of Pediatric Oncology (PSPO) where difficult and complex cases are being discussed. Recently, for uniformity of staging and treatment we have proposed nationwide protocol of RB under leadership of PSPO which is to be adopted by all oncology centres and ophthalmologists of the country, the results of which are yet to be determined.

## Conclusion

Risk stratification based on histopathological features of the enucleated eye is an effective way of tailoring adjuvant therapy. Significant number of patients avoided chemotherapy with very low risk of relapse. Globe salvage in advanced disease is possible with the introduction of newest modalities like IAC, but it has limited availability due to cost effectiveness and lack of expertise in resource limited setting. Hence, in advanced unilateral RBs, we recommend upfront enucleation for risk stratification and treatment accordingly in our setting.

## Conflicts of interest

The authors declare no conflict of interest.

## Disclosure

The abstract was presented as an e-poster at SIOP 2021.

## Funding

None.

## Figures and Tables

**Table 1.** Patient and tumour characteristics (*n* = 113).

Patient characteristics	*n* (Percentages %)
**Mean age at presentation**	37.4 ± 24.5 months
**Gender**	
**Male**	65 (57.5%)
**Female**	48 (42.5%)
**Presenting signs** ^a^	
**Leukocoria**	84 (74.3%)
**Proptosis**	11 (9.7%)
**Red eye**	6 (5.3%)
**Vision loss**	7 (6.2%)
**Squint**	3 (2.7%)
**Ptosis**	1 (0.9%)
**IIRC Group**	
**Group C**	5 (4.4%)
**Group D**	68 (60.2%)
**Group E**	40 (35.4%)
**Pathology**	
**Anterior segment invasion**	38 (33.6%)
**Ciliary body invasion**	16 (14.1%)
**Massive choroidal invasion**	20 (17.7%)
**Scleral invasion**	12 (10.6%)
**Pre-laminar invasion**	15 (13.3%)
**Post-Laminar invasion**	33 (29.2%)
**Optic nerve cut end invasion**	29 (25.7%)
**4-year OS**	74%
**4-year EFS**	71%

a One patient had strong family history, had upfront enucleation despite having Group C

**Table 2.** Outcomes of disease regarding histopathological risk stratification.

	Outcomes					
Risk group based on histopathological features (*n* = 113)	Complete response	Disease relapse	Disease progression	Death	Abandonment	*p* value
**Low risk**	29	1	0	0	0	<0.05
**Intermediate risk**	43	6	1	1	3	
**High risk**	11	5	6	3	4	
